# Technology solutionism in paediatric intensive care: clinicians’ perspectives of bioethical considerations

**DOI:** 10.1186/s12910-023-00937-6

**Published:** 2023-07-28

**Authors:** Denise Alexander, Mary Quirke, Carmel Doyle, Katie Hill, Kate Masterson, Maria Brenner

**Affiliations:** 1 School of Nursing, Midwifery & Health Systems, University College Dublin, Dublin, Ireland; 2 School of Nursing & Midwifery, Trinity College Dublin, Dublin, Ireland

**Keywords:** Bioethics, Child, Clinical technology, Complex care, Long-term ventilation

## Abstract

**Background:**

The use of long-term life-sustaining technology for children improves survival rates in paediatric intensive care units (PICUs), but it may also increase long-term morbidity. One example of this is children who are dependent on invasive long-term ventilation. Clinicians caring for these children navigate an increasing array of ethical complexities. This study looks at the meaning clinicians give to the bioethical considerations associated with the availability of increasingly sophisticated technology.

**Methods:**

A hermeneutic phenomenological exploration of the experiences of clinicians in deciding whether to initiate invasive long-term ventilation in children took place, via unstructured interviews. Data were analysed to gain insight into the lived experiences of clinicians. Participants were from PICUs, or closely allied to the care of children in PICUs, in four countries.

**Results:**

Three themes developed from the data that portray the experiences of the clinicians: forming and managing relationships with parents and other clinicians considering, or using, life sustaining technology; the responsibility for moral and professional integrity in the use of technology; and keeping up with technological developments, and the resulting ethical and moral considerations.

**Discussion:**

There are many benefits of the availability of long-term life-sustaining technology for a child, however, clinicians must also consider increasingly complex ethical dilemmas. Bioethical norms are adapting to aid clinicians, but challenges remain.

**Conclusion:**

During a time of technological solutionism, more needs to be understood about the influences on the initiation of invasive long-term ventilation for a child. Further research to better understand how clinicians, and bioethics services, support care delivery may positively impact this arena of health care.

**Supplementary Information:**

The online version contains supplementary material available at 10.1186/s12910-023-00937-6.

## Introduction

Technological advances are a key feature of paediatric intensive care, particularly life-sustaining technology, which has become increasingly sophisticated and available [1–4]. Long-term life-sustaining technology for children is initiated on many more occasions than in previous decades [5], and has become the subject of ethical, medical and social debate [3, 6–9]. The increased survival rates of children in paediatric intensive care units (PICUs) is often accompanied by an increase in long-term morbidity [10]. As a result, there are complex and dynamic issues and arguments around its potential, and its limitations [3, 6, 7, 9]. New technologies may offer an increased lifespan for a child, as well as the opportunity for growth and development, and for a child to move from hospital to home. We are in a time of significant medical advancement and the long-term implications of the technology on the child, both positive and negative, are only beginning to be evidenced [3, 11, 12], often with uncertainty of outcomes for this cohort of young people. It is in this context that clinicians in PICUs must make decisions about the initiation of long-term technology to sustain a child’s life. The concept of technology dependence itself refers to “a wide range of clinical technology to support biological functioning across a dependency continuum, for a range of clinical conditions. It is commonly initiated within a complex biopsychosocial context and has wide-ranging sequelae for the child and family, and health and social care delivery” [5, 13]. This study, part of a larger project, specifically explored the meaning given by clinicians to the bioethical issues associated with increasingly sophisticated and available long-term use of technology that can sustain the life of a child, using invasive long-term ventilation (I-LTV) as an exemplar of technology dependence.

## Methods

### Sampling and participants

Four international sites in Australia, Ireland, the Netherlands and the United States took part in the study. Within these countries, hospitals acted as gatekeepers to participants. These countries were chosen to represent, as far as possible, a variety of health systems, and wider environments. Participants were recruited via the local gatekeeper, and snowball sampling identified further clinicians who met the inclusion criteria [14]. Clinicians were eligible to participate if they worked in PICU or an adjacent area with experience caring for children who required I-LTV as an example of technology dependence. We placed no limits on our sample size, but we aimed to interview all the participants who wished to share their lived experiences.

### Data collection

Each participant took part in an unstructured interview with one of the six interviewers in the research team, all the interviews were in English. The interviewers focused on geographical areas, for example two interviewers focused on Australia, two on the Netherlands and Ireland, and two on the United States. In addition to this, a pragmatic approach was taken so that interviewers could schedule interviews with participants from any country according to their availability. In the interview, the participants were asked to recall a recent experience they had of a child who needed, or who had, I-LTV initiated to sustain life. We used a standard question to start the conversation: “Please tell me about your experience of initiating technology dependence to sustain a child’s life, for example I-LTV” and asked them to give a recent example of a child they had cared for. The researchers prompted interviewees to expand on any points they mentioned. Interviews were conducted remotely via video conferencing between April 2020 and November 2020. Each interview lasted between 35 min and an hour.

### Data analysis

This study was a phenomenological investigation, which explored how individuals uniquely experience and interpret the world of the PICU. A central feature of phenomenology is that the interviewer focuses on the experience of the participant, and reduces the researcher’s input to a minimum, in order to avoid bias as much as possible. As such we focused on the rich descriptions of the lived experience provided by the participants [15]. The conceptual framework we used is based on the work of Van Manen [16, 17]. The transcribed interviews were imported into the software program NVIVO [18] to help organise and refine the data. Data were read and coded in line with the analysis technique described by Van Manen [16, 17].

### Rigour

The criteria of rigour described by Guba and Lincoln [19] guided this research. Interview protocols were based on existing literature in the area [5, 13], and site gatekeepers guided purposive sampling of participants to maximise the relevance of the findings to those working with children in need of technology dependence to sustain life. Six researchers conducted the interviews and communicated frequently within the research team during that process. All methodological steps and methods were documented and agreed by all researchers. The organisation of the raw data and emerging findings were documented and discussed regularly within the team.

### Ethics

Ethical approval was obtained from the research ethics committee in the host institution, and subsequently by the research ethics’ committees in each of the international sites for the project. Potential participants were emailed information about the purpose of the study via a gatekeeper. Individuals then contacted the research team directly if interested in taking part in an interview. Participants were provided with an information leaflet and completed an electronic consent form via Qualtrics [20] prior to taking part in the study. Interviews were conducted remotely via Zoom [21] at a time of the participants’ convenience. All collected data were confidential and stored securely in line with the host institution’s data protection guidelines. Transcriptions were made from the recordings of the interviews, and then anonymised before the interview audio files were deleted.

## Results

### Sample description

Seventy-eight clinicians took part in the study. Clinicians comprised physicians (*n* = 40), nurses (*n* = 26) and other health professionals (*n* = 12) who are members of the wider multidisciplinary team including physiotherapists, medical social workers and clinical bioethicists. The majority of clinicians were based in Australia (*n* = 28), followed by Ireland (*n* = 19), the United States (*n* = 16) and the Netherlands (*n* = 15).

**Fig. 1:**
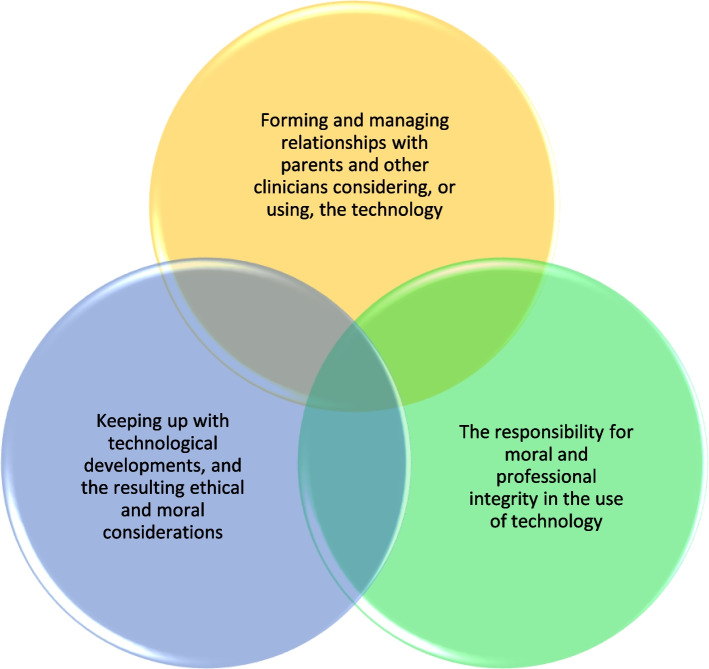
Bioethical issues in the context of life-sustaining technology for children

Three themes were developed from the data that reflect the phenomenon of working in an environment of fast-moving technological developments in a PICU that can sustain the life of a child. These were: Forming and managing relationships with parents and other clinicians considering or using the technology; the responsibility for moral and professional integrity in the use of technology; and keeping up with technological developments, and the resulting ethical and moral considerations. The themes were closely related to each other and build up a picture of the perceptions of the clinicians of the bioethical considerations that surround the initiation of life-sustaining technology in a child. The interaction of the three themes is illustrated in Fig. 1.

#### Forming and managing relationships with parents and other clinicians considering, or using, the technology

The clinicians valued a good relationship with the family of the sick child. The ethical issues surrounding the initiation of technology dependence are often not straightforward, and understanding the values and motivations of all involved are important:

“I think one of the things we try very hard as an intensive care faculty to do is to own the process of helping families make decisions with families rather than letting it be driven by consultants …like ‘I can do this technically and that will keep your child alive’, that’s different than saying ‘is this what you want?’ ” (Neonatologist, United States).

The technology facilitates some goals of care conversations, leading to deeper discussions about the child’s survival or future quality of life, and it can be challenging to discuss. Communicating effectively about the availability and particularly the limits of technology can, on occasion, be problematic. Some clinicians believed that relationship-building between themselves and the parents suffers because parents are increasingly aware of what technology is available, and they expect that it should be used for their child, but that the uncertainty of the outcome, or the utility of the technology over a long period of time is difficult to communicate.

In general, clinicians interviewed valued the contribution of an ethics consultation. They saw the bioethics service as helping them to identify and express all relevant aspects of an issue, including difficulties with prognosis, perspectives on quality of life, and different values and expectations of the technology held by all parties to the discussion. As part of the relationship with the family, the clinicians discussed how, on occasion, they had to explain why a treatment is not available, or unsuitable, for their child. If a family cannot understand why clinicians do not recommend technology dependence for their child, the situation was seen by the clinicians as potentially challenging: “it’s a tricky road sometimes” (Specialist consultant, Ireland), and this leads to possible debate and disagreement.

Some participants stated that families explore and determine what is possible for their child before coming to a particular hospital in their country, and are as a result resolved in their wishes for a particular care pathway. They felt this made relationships between the clinicians and families challenging, particularly if the clinicians in the referring hospital request a specific course of action:

“So what I would say is that in the last few years we’re seeing a lot more of that conversation happening before we even see the babies and the families. So we’re being sent children now, infants who are just, the decision has already been made before they get here: ‘we would like you to perform a tracheostomy and a gastrostomy tube on this patient and send them to rehab’ ” (Neonatologist, United States).

It is often beneficial for families when they form relationships with other families in similar situations in PICU. However, on occasion this may make the clinicians’ communication with the parents harder as there can be misinformation because each case is unique: “there’s a lot of nuances that families may not totally understand” (Specialist consultant, Ireland).

#### The responsibility for moral and professional integrity in the use of technology

The clinicians expressed feelings of professional and moral responsibility as important factors in the consideration of the ethics of initiating I-LTV. Clinicians felt a responsibility for the immediate health and welfare of the child, and also for the future consequences of any decision that may be made in respect of their care. In general, they strove to offer realistic options, but expressed on occasion that this was complicated by what they felt were the competing responsibilities that emerge because of the availability of the technology: for example the desire to preserve life, but also not to extend the life of a child who would not improve, or even experience pain as a result of the technology, and any possible adverse effects on the wider family into the future. Clinicians stated that differing priorities and concepts of wellness or quality of life need to be discussed and understood by all of those involved, as the meaning of quality of life can be substantively different to different people. The influences of organisational demands, resources, and in some cases the impossibility of accurate prognosis for a child using new technology need consideration alongside the moral and professional concerns.

Clinicians reflected that the treatment must be in the best interest of the individual child. They recognised that this term is itself very subjective, and valued the input of bioethical discussions around what they felt was the best interest, particularly if this view differed from that of the parents. In some cases, the ‘best interests’ of the child is not felt to be served by initiating long-term life-sustaining technology: “we are certainly not anti-support however there are families that are not offered non-invasive or invasive support because it’s felt to be either inappropriate or unhelpful … we are not obligated to bring up things that we wouldn't offer” (Intensivist, Australia).

In many cases it is very difficult to come to a conclusion as to what is the best course of action, and the support of other health professionals was also felt to be invaluable: “if you are unsure [about a course of action] and there’s a very strong-willed parent I would always get another opinion” (MDT, Ireland).

There are ethical dilemmas that require consideration when technology is initiated for a child who the clinicians feel will not improve or benefit from the technology. This may occur if the family wishes that technology be initiated, or there is a felt pressure to provide what technology is available. An example of this was given when I-LTV was initiated for a child with a genetic disorder, who had little chance of improvement or even comfort as a result, despite life being sustained: “this poor child was suffering every day” (Intensivist, Australia). A member of the MDT stated:

“very experienced staff nurses were saying to me… ‘I can’t do this anymore’; ‘what are we doing?’ The child was put through a lot and I suppose in terms of the technology side I suppose there were two very, very serious conditions coexisting that you don’t see coexisting” (MDT, Ireland).

Clinicians reported that it has become easier to initiate technology dependence, because of a relaxation of the rules of eligibility, and because of improvements in the technology. In many cases, this is beneficial and leads to innovations that improve the quality of life of a child using the technology, such as the development of increasingly quieter and more portable ventilators. However, some clinicians felt that the developments resulted in fewer opportunities to explore the ethical and practical ramifications of an individual treatment decision:

“… but of course the envelope has been pushed and pushed and whether or not that pushing of the envelope is in terms of the use of technology, when it was otherwise not considered to be appropriate, is coming from clinicians who want to try it out, or coming from families to say ‘we know this exists … why don’t we at least keep our child alive on this bit of technology’” (MDT, Australia).

The level of clinical responsibility was sometimes referred to by the clinicians as a burden as they described the gravity of the decisions they are involved in. One aspect of responsibility in initiating I-LTV for a child lies in attempting to impart the full picture to the parents. The clinicians stated that they had concerns that the promise of the technology would not live up to parental expectations, yet recognising that for the parents “I guess it’s a really vulnerable time, right” (Nurse, Australia). The focus for the clinicians is sometimes more about trying to manage parental expectations towards a realistic prospect of what the technology will mean for the child and family:

“A lot depends upon how things are presented to families, and if something like tracheostomy is presented in a way, as a cure without adequate discussion of the discomforts and burdens of the intervention, it doesn’t give the whole picture and the context is extremely important” (Complex care paediatrician, Australia).

The clinicians describe the importance of their previous experiences in informing future care decisions. The fact that many of these children are repeatedly seen in PICUs – “we see routinely … the technology dependent kids over and over again” (Intensivist, United States) suggests that some of the clinicians felt that although “the medicine is so good, we can save those who ten or fifteen years ago would have died from prematurity or known chromosomal disorders” (Intensivist, United States), the technology may sustain life, but morbidities may occur, and the technology will not solve all the issues. One nurse expressed her emotions about a particular patient:

“And it was obvious after six months that [the baby] was never going to get off a ventilator. And I think [the baby] was on full ventilation trying to manage at home and I think it was a really awful experience for the family…” (Nurse, Ireland)

The clinicians also recognised the difficulty of objectively assessing the quality of life of a child and of a family; a situation that leads to complex ethical quandaries:

“I’m not sure if all [of the families] presented with that same question 10 years later they would all feel the same way about it” (Respiratory specialist, United States).

The ethical issue concerning the different levels of resources available in the community, such as home nursing provision, is also of concern to some of the clinicians: “that’s what we see is a major problem” (Nurse practitioner, United States). This is also a concern in the light of the increasingly long-term nature of some of these supports, a phenomenon that has not been seen before for these children:

“it’s very difficult to transition our patients to adult institutions because the supports aren’t within the adult systems to provide the same type of care that we do in paediatrics” (Nurse, United States).

#### Keeping up with technological developments, and the resulting ethical and moral considerations

Many clinicians discussed the increased availability, affordability, and the relative ease of eligibility for life-sustaining technology in recent years: “[it has] increased exponentially really from when I first started” (Nurse, Australia). As a result, there is a perception of “greater acceptance of technology” (Specialist consultant, Australia) on an institutional level and greater expectations that technology will be used by the public: “maybe that’s our society here, but it’s not ethically justifiable not to offer” (Nurse, United States). Some clinicians see technology as having a positive impact not only on survival, but on the quality of life of some children:

“I see people living, like good lives, happy lives and the technology is, it’s there, it’s a bit annoying but It’s part of their lives” (MDT, Australia).

Other clinicians worry about the long-term consequences of using life-sustaining technology in some children, which are difficult to predict, and may have unintended consequences depending on age and disease:

“I’ve got children I’ve followed for 20 years, who went home with technology. What we’re finding also is that the body wasn’t meant for a[long-term] tracheostomy tube and positive pressure ventilation and we’re finding now in children that have been on a ventilator for 20 years, 25 years, that we’re having significant medical problems keeping them alive with airway difficulties” (MDT, United States).

What is clear from the interviews is that there is much greater expectation that technology will be used:

“I think that nowadays you’re seeing a practice, paradigm shift where ok the baby is at three months of age, still on a ventilator and can’t feed, this is what we have to offer, this is what we can do next. And so the hospitals that would send us these babies with a question mark are now saying this is now the paradigm that we should pursue unless a family strongly pushes back against that.” (Neonatologist, United States)

To keep up with developments and use of life-sustaining technology more effectively, some suggested the need for more training in terms of managing expectations and prognoses, either from bioethics or from other sources. In some cases, the possibilities and the consequences of technology are yet to be fully articulated:

“So the medicine is so good then there has to be better education for staff and trainees about how we approach families about [life-sustaining] technology” (Intensivist, United States).

One clinician expressed a worry about the deskilling of health professionals as a result of more sophisticated technology which seems to have an element of independence from the prescribing physician and even from the child. In addition, as technologies are used more frequently by families at home, they are being used in many more ways than from their original purpose. One member of the MDT gave an example of this:

“I’ve seen dads of families who will rig up, against our advice mind you, will rig up all sorts of things to make it easy to travel with oxygen from one place to grandma’s place and whatever. To take the ventilator, to keep humidifying in the car when there’s not a normal way of doing that but they want that, they believe that, so they go and do that (MDT, Australia).

This can create a sense of unease on the part of the clinicians, who are unable to monitor the use of equipment outside of the hospital environment:

“we’ve had a few cases where, especially complex kids who require all sorts of you know care, who are using technology at home. And that’s probably where I was thinking where parents start not using it appropriately or using it in their own way or where it’s far too complex to be having that technology in the home” (MDT, Australia).

As a consequence, the need for increased support from bioethics professionals was required:

“…there’s much more ethical considerations to be had, just because we have the technology doesn’t necessarily mean there’s a resource in the community to support these families … just to help you make your decisions, I think would be huge. I think it’s going to become very necessary” (Specialist consultant, Ireland).

Some participants felt there was, at times, an institutional or even a cultural push to be “more aggressive with treatment” (MDT, United States). This may be self-perpetuation because of its availability and information about the technology. The question seems to be whether it is the clinicians extending the limits of what is treatable, or the technology development that is pushing the barriers of what is possible in medicine, and to be “more cutting edge” (MDT, United States). This situation can create a cycle that is increasingly difficult to challenge. This, in ethically difficult situations, can become a source of stress if the clinical decision in the child’s best interest does not ally with the administration’s ethos.

## Discussion

Our research shows how the topic of life-sustaining technology in children may bring up uncomfortable questions about what life means, and what constitutes a good quality of life in the children whose life is sustained by means of I-LTV. The power of technology to sustain life is undeniable and in many cases undoubtedly perceived as miraculous. However, this research has also shown that, in the lived experience of the clinicians we interviewed, setting those many benefits aside, the practical and ethical challenges that surround the initiation of technology dependence is urgently in need of articulation and this was the topic at the forefront of clinicians’ minds when describing their lived experiences of considering the initiation of life-sustaining technology for a child.

The ethical uncertainties of rapid technological developments and therapies leave many clinicians struggling to make decisions, resolve conflicts or be at ease with some of the care plans that have been made for children under their care, whatever approach or decision that plan may entail. Deciding how to proceed in situations where technology dependence is required to sustain life is often very difficult due to the uniqueness of each case [22]. Conflicts may emerge in these profoundly difficult decisions because of the many unknowns in the situation, and are indicative of the extent to which value judgements influence decisions. This, combined with including the different perspectives of what is an appropriate ethical response in the presence of life sustaining technology [23] can be very challenging to overcome where consensus by all involved is desired. Some of our participants discussed the control of technology in the home, and their reservations about simultaneously being responsible for the technology; but also the parents’ growing expertise in using it, and adaptation of the technology to the everyday life of their child, an issue discussed by Toly et al. [24].

Differences in moral perspectives are real and legitimate, and our research illustrates the struggles of clinicians who, in their own views, aim to provide the most appropriate care pathway for a child, while also respecting views that differ from their own when they arise. Socio-political and socio-cultural influences may also be present, for example in some countries the focus is on the rights of the child, and in others, the rights of the family are paramount. An important finding of this research is how bioethical services are perceived as helping clinicians and parents to recognise, consider and debate different strongly held points of view. Whilst not offering a panacea for those involved in decision-making at this critical juncture, the literature shows that bioethics as a profession has recognised these changing parameters in terms of their role in supporting clinicians. Decisions are made in the light of a “myriad of complex social, personal and medical factors” [25] that influence the process. It is recognised that bioethics in the context of life-sustaining technology is a “new and burgeoning field” [25], and one in which professional and lay attitudes may differ, and indeed any investigation into this issue is unlikely itself to be without implicit or explicit bias. Recent national controversies about child survival and withdrawal of technological dependence demonstrate the challenge facing clinicians and parents; often compounded by the difficulty of prognosis for children whose lives are sustained by technological devices. Brick et al. [26] found a substantial difference between the analysis of medical ethicists and the views of the general public, in what would be the best ethical solution to withdrawing or withholding a life sustaining technology such as ventilation. Larcher et al. [4] state that there is no single ethical framework that embraces all views on questions of technology dependence in children to sustain life; but suggests that certain fundamental considerations be addressed. These are the duty of care to the child from health professionals and from parents; the need to respect the rights of the child; and the requirements of the law [4]. The ethical frameworks used to help understand decisions and resolve such dilemmas have had to adapt as a result of the increase of technological interventions for life-limiting conditions.

Traditionally, before any treatment can begin, clear medical indication that the treatment has a reasonable chance of providing benefit and is unlikely to cause harm must be present; and the patient’s advocate, such as the parent, must give informed consent [27]. However, in cases where life can only be sustained by the initiation of technology dependence, the balance of risk and benefit and the existence of truly informed consent are not so clear. Our participants expressed how difficult it was to impart the consequences of a course of action to parents, a challenge also described by Welie et al. [27]. Ensuring that a patient or a parent understands the possible long-term consequences of life-sustaining technological treatment alongside the consequences of choosing not to initiate can be difficult [4]. When it is obvious that a new technology cannot restore health and may even cause suffering it seems inevitable that decisions around whether or not to initiate technology dependence involves value judgements, we can see this in our own study when clinicians describe the ‘suffering’ of a child or the distress of the family. These value judgements will be unique to each case under discussion. The increased involvement of families in the decision-making process arguably makes the need for ethical guidance more important [28].

New technological innovations mean that the clinicians’ perspective of the role of bioethics changes, moving, for example, to help clinicians understand and appreciate the parents’ reasoning they do not initially accept. The concept of a threshold view is sometimes used to guide these decisions; where an upper and lower threshold of treatment is clinically defined (for example, treatment will definitely help the child, or treatment will be of no benefit), and within these spaces decisions are more generally in terms of the cultural norms of that institution and country [22, 29]. If the child’s prognosis is uncertain between these two zones, then either choice, of initiating or not initiating technology dependence, is ethically permissible. As Brick et al. [26] state, this recognises the impossibility of certainty of prognosis, and the interests of family members in terms of burden of care for the child.

## Limitations

In any qualitative study, there is the risk of bias in data collection and in interpretation. Our data collection was necessarily constrained by our access to clinicians who were willing to participate, and were invited to do so by gatekeepers in our four established global sites. The majority of our participants were physicians, which reflects the individuals who are most likely to make decisions alongside patients and parents; the views of other clinicians such as nurses, and members of allied health professions are not so numerous in the study, but nevertheless contribute to our understanding. This study has only looked at the perspectives of the clinicians on the subject of initiating technology dependence in an age when life-sustaining technology is increasingly available and expected by many. It is important to consider that the views of the clinicians, while strongly held, are only part of the story, and the views of the families, or the children who are technology dependent are not described in this work.

We asked clinicians to tell us about their experiences of initiating technology dependence to sustain a child’s life. The discussions that resulted in this portrayal of the lived experiences of the clinicians focused on more challenging cases, for example where the clinicians felt that technology was not in the child’s best interests, or there was disagreement with the family as to the best course of action, and a certain level of moral distress was experienced by the clinician caring for a child. The stories of children and families who are thriving on technology dependence are largely absent from this research. It is known that clinicians’ perspectives may differ from those of the families themselves [30, 31], and that the voices of the families who, because of the stability of their ventilated child, are not known to PICU clinicians may be absent from this discussion. It is important to consider that the lived experiences of the clinicians are only part of the story.

## Conclusion

The current possibilities to sustain a life and transform quality of life for some, and the implications of this for the delivery of care in the acute and community setting, has given rise to new ethical and clinical considerations. This is a time of technological change, and of change in the way decisions can be made and how they are made. This work has shown that more needs to be understood about the influences on the initiation of technology dependence, and the role of the technology itself in this process. Further research that provides a greater understanding of how the bioethics services work with clinicians in order to provide the comprehensive support in the context of increasingly sophisticated life-sustaining technology will positively impact this arena of healthcare.

### Supplementary Information

**Additional File 1: Interview Guide.**

## Data Availability

The qualitative data is not available to uphold the confidential nature of this research approach.
