# Correction: Technology solutionism in paediatric intensive care: clinicians’ perspectives of bioethical considerations

**DOI:** 10.1186/s12910-023-00946-5

**Published:** 2023-08-28

**Authors:** Denise Alexander, Mary Quirke, Carmel Doyle, Katie Hill, Kate Masterson, Maria Brenner

**Affiliations:** 1 School of Nursing, Midwifery & Health Systems, University College Dublin, Dublin, Ireland; 2 School of Nursing & Midwifery, Trinity College Dublin, Dublin, Ireland

Following publication of the original article [1], in this article ref. 5 and 13 was incorrect and should have been read as follow:

5. Alexander D, Eustace-Cook J, Brenner M. Approaches to the initiation of life-sustaining technology in children: a scoping review of changes over time. J Child Health Care. 2021;25(4):509–22. 10.1177/1367493520961884.

13. Alexander D, Quirke M, Berry J et al. Initiating technology dependence to sustain a child’s life: a systematic review of reasons. J Med Ethics. 2021. 10.1136/medethics-2020-107099.

The original article has been corrected.
