# Supplementary material for: Technology solutionism in paediatric intensive care: clinicians’ perspectives of bioethical considerations
Source: BMC Med Ethics. 2023 Jul 28;24:55. doi: 10.1186/s12910-023-00937-6 (PMC10386660; doi:10.1186/s12910-023-00937-6)
Supplement: Supplementary file 1 — Additional File 1: Interview Guide. [file 12910_2023_937_MOESM1_ESM.docx]

## Appendix 1: Interview Guide

| INTERVIEW | | |
| --- | --- | --- |
| Immediately before interview begins | Check for consent.  Check that they are aware of any local supports they may need, and how to contact them if necessary.  Introductions and general conversation.  Give summary of study, aim of interview and opportunity to ask questions.  Inform participant that interview will be recorded, begin recording.  Reiterate privacy and confidentiality and reiterate the opportunity to discontinue the interview at any point.  Demographic questions: age, number of years on bioethics committee, any other roles in addition to their role on the ethics committee, professional background |  |
| During interview | **Please can you tell me about your experience of working with clinicians when initiating invasive long-term ventilation to sustain a child’s life?** |  |
